# Orchestration of Adaptive T Cell Responses by Neutrophil Granule Contents

**DOI:** 10.1155/2019/8968943

**Published:** 2019-03-10

**Authors:** Danielle Minns, Katie Jane Smith, Emily Gwyer Findlay

**Affiliations:** Centre for Inflammation Research, School of Medicine and Veterinary Medicine, University of Edinburgh, Edinburgh EH16 4TJ, UK

## Abstract

Neutrophils are the most abundant leukocytes in peripheral blood and respond rapidly to danger, infiltrating tissues within minutes of infectious or sterile injury. Neutrophils were long thought of as simple killers, but now we recognise them as responsive cells able to adapt to inflammation and orchestrate subsequent events with some sophistication. Here, we discuss how these rapid responders release mediators which influence later adaptive T cell immunity through influences on DC priming and directly on the T cells themselves. We consider how the release of granule contents by neutrophils—through NETosis or degranulation—is one way in which the innate immune system directs the phenotype of the adaptive immune response.

## 1. Neutrophils Are Sophisticated Cells Able to Adapt to Changing Inflammation

Neutrophils are not simple bags of enzymes sent to kill pathogens before the adaptive immune cells move in. In fact, they are able to respond to altered inflammatory status; neutrophils can produce cytokines [1], alter their gene expression during inflammation [2] and throughout “aging” [3], and survive for significantly longer than traditionally thought, with one study placing lifespan from bone marrow at 5.4 days [4]. As a consequence, neutrophils are able to adapt to changing conditions and direct other cells' behaviour—a task which they can perform with some sophistication.

## 2. The Adaptive T Cell Response Generates in the Presence of Neutrophil Mediators

As adaptive immune responses develop, T cells are primed by dendritic cells (DCs) in the lymph node and proliferate *in situ* before moving into the tissue where their antigen of interest is situated. Here, they encounter antigen, receive additional signals from antigen-presenting cells (APCs) and local cytokines, and carry out their effector functions. However, this response does not happen in isolation. The DCs scanning for antigen in the respiratory tract during influenza infection, for example, also encounter millions of neutrophils, which can out number them manyfold [5, 6], as do the influenza-specific T cells which subsequently leave the lymph node at the peak of inflammation. As these neutrophils will be degranulating, dying, and producing extracellular traps (NETs, [7]), the DC and T cells are in effect moving into a soup of neutrophil-produced inflammatory mediators. It is no surprise that these mediators have profound effects on T cell differentiation, survival, proliferation, and effector function.

In this review, we will consider how the granule contents released during neutrophil degranulation and NETosis affect the development of adaptive T cell responses. We are discussing extruded mediators only, and not the antigen-presentation capacities or cell-cell interactions performed by neutrophils nor the outcomes of whole apoptotic or necrotic neutrophils being engulfed. It is our contention that the inflammatory mediators released by neutrophils allow these innate cells to exert some control over the tissue environment and direct later adaptive immune responses.

## 3. Conflicting Data on How Neutrophils Affect T Cell Responses

**Figure 1:**
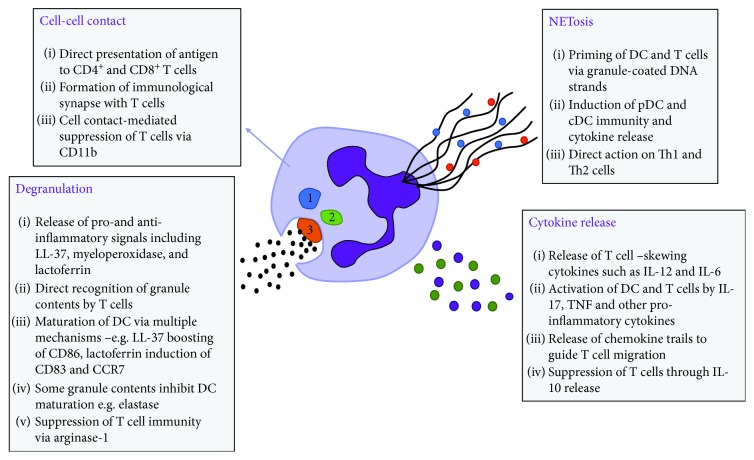
Neutrophils impact T cell immunity through many methods. Neutrophils undergo cell-cell contact, NETosis, degranulation, and cytokine release which affects T cell responses by either activating or suppressing their function. (1) Primary granules, (2) secondary granules, and (3) tertiary granules.

Recent years have seen an explosion of research into how neutrophils affect DC and T cell biology; however, these data are confusing, with neutrophils either suppressing or promoting T cell activation depending on the context (Figure 1).

There are a number of murine models in which the T cell responses can be exacerbated by depleting neutrophils, implying they have a regulatory role [8, 9]. This suppression of T cell responses by neutrophils requires close contact and development of an immunological synapse [10, 11]—perhaps as the mediators thought responsible, reactive oxygen species and H_2_O_2_ do not diffuse far. Uptake of apoptotic or necrotic neutrophils also inhibits DC antigen presentation and co-stimulation, resulting in reduced T cell responses—a situation which can be exploited by pathogens. For example, neutrophils capture *L. major* and are subsequently engulfed by DCs, suppressing antigen presentation and T cell priming [12, 13].

In the second group of research, neutrophils are proinflammatory and promote T cell responses. Neutrophils induce the migration, maturation, proinflammatory cytokine production, and priming capabilities of DCs through contact- and cytokine/chemokine-dependent mechanisms [14–23]. Depletion of neutrophils in mouse models of inflammatory disease leads to decreases in virus-specific CD8^+^ T cell responses [24] and a lack of skewing to protective subsets [25, 26]. Further, neutrophils can directly present antigen to T cells and directly stimulate T cell proliferation in response to superantigen [27–31].

It is not yet clear why and how these two situations exist. It may reflect the presence of different populations of neutrophils [32] which are not yet stratified; for example, the maturation status of neutrophils released from the bone marrow during sepsis, which are suppressive to T cells [10], differs markedly from those present in the highly inflammatory environment of hyperlipidemia [33], which are primed and produce high levels of myeloperoxidase. In addition, the ability of neutrophils to alter their expression of surface molecules and to transcribe RNA once they have left the bone marrow means the generation of localised populations of cells may well have differential effects on adaptive immunity (reviewed in [34]).

Other possibilities are that the different effects noted are a consequence of the inflammatory model or infection used or of particular features of dendritic cell or T cell biology that are only expressed in certain inflammatory states; the situation may also reflect different experimental techniques, particularly for the isolation of neutrophils (see Section 5 - a note on immature and low density neutrophils). Further, murine and human neutrophils do differ in many ways (reviewed in [35]) and use of mouse models must be considered as a variable.

In this review, we attempt to discuss a number of ways in which neutrophil granule contents affect priming, differentiation, and survival of *αβ* T cells. These granule peptides also show a variety of effects, with both suppressive and activatory impacts on T cell immunity—we discuss the complexity of these responses.

## 4. Impact of Neutrophil Granule Contents of DC-T Cell Immunity

### 4.1. Degranulation

Degranulation is the exocytosis of antimicrobial or cytotoxic molecules from intracellular granules, which functions as a defence mechanism to kill invading microbes. Neutrophils are activated and undergo degranulation through a variety of ligands (including LPS, IL-8, fMLF, and C5a) binding to cell-surface receptors which include G protein-coupled receptors (GPCR), Fc-receptors (Fc-R), pattern recognition receptors (PRRs), and chemokine receptors [36–38].

Following neutrophil activation, different granules possess distinct propensities for extracellular release. Neutrophil granules are divided into different subsets—primary (azurophilic), secondary (secretory), and tertiary (gelatinase) granules in addition to secretory vesicles (SVs). The different granules are formed sequentially during neutrophil differentiation and are released in the opposite order; the timing of synthesis determines the sequence in which the granules are released [39–42]. The strict hierarchy of release is secretory vesicles then tertiary, secondary, and finally primary granules [37].

Proteomic analysis delineating granule contents [43, 44] reveals that primary granules contain myeloperoxidase (MPO), serine proteases cathepsin G and neutrophil elastase (NE), and alpha defensins; secondary granules contain antimicrobial proteins and peptides including lactoferrin and cathelicidin (hCAP-18), pentraxin and haptoglobin, and some metalloproteinases (MMPs); finally, tertiary granules contain gelatinase, lysozyme, and some MMPs.

In this section, we discuss the contribution of components of these granules to T cell immunity.  Table 1 lists a summary of results for prominent mediators from each granule.

#### 4.1.1. Myeloperoxidase

MPO is a 150 kDa cationic dimeric protein which produces hypochlorous acid from H_2_O_2_ and chloride during the respiratory burst; it is present in the primary granules and is an important antimicrobial agent [45, 46].

MPO-specific CD4^+^ T cells are present in patients with anti-MPO glomerulonephritis (GN) [47–49]; these can mediate glomerular injury directly as they recognise MPO released from neutrophils undergoing degranulation, apoptosis, and NETosis, inducing [50] delayed-type hypersensitivity through enhanced IFN-*γ* production. Immunising mice with MPO in this model leads to increased infiltration of CD4^+^ T cells [48]. Likewise, direct injection of MPO during a model of anti-MPO glomerulonephritis induced significant IL-17A production and development of an MPO-specific DTH response as well as renal disease [51]. Therefore, in this particular inflammatory disease, MPO induces strongly proinflammatory T cell responses owing to it being an autoantigen.

However, in other forms of glomerulonephritis where MPO is not an autoantigen (including antiglomerular basement membrane GN and pristine-induced lupus nephritis), endogenous MPO suppresses T cell responses. Here, *MPO^−/−^* mice showed increased CD4^+^ T cell accumulation and proliferation suggesting MPO attenuates T cell responses [52, 53]. MPO has profoundly suppressive effects on DC and CD4^+^ T cells *in vitro* and *in vivo*. MPO inhibits ConA-induced proliferation of human T cells *in vitro* [54], and CD4^+^ T cells have increased activation, proliferation, and proinflammatory cytokine production in *MPO^−/−^* mice [55]. This was mediated by neutrophils depositing MPO in lymph nodes, where it decreases DC migration and activation. In agreement with this, mice lacking MPO have enhanced T cell immunity and more severe antigen-induced arthritis [55].

In addition, the reactive intermediate taurine chloramine, formed as a result of MPO-catalyzed reactions, inhibits the release of TNF-*α*, IL-6, and IL-12 from murine DC and inhibits the capacity of DC to induce the release of IL-2 and IL-10 from T cells *in vitro* therefore suppressing T cell activity [56, 57].

It is important to note that the majority of work demonstrating a proinflammatory T cell response to MPO has been performed in patients with anti-MPO glomerulonephritis and vasculitis, who present with ANCA (antineutrophil cytoplasmic antibodies) which react to proteinase 3 and MPO in particular; this disease may involve unusual mechanisms of action of MPO not found in healthy individuals including it acting as an autoantigen.

#### 4.1.2. Alpha Defensins

The human neutrophil peptides (HNP1-3), also known as *α*-defensins, are small (c.4 kDa), cationic, amphipathic antimicrobial peptides which are highly abundant in neutrophils, comprising 30-50% of the protein content of primary granules; they are not present in mouse neutrophils (entertainingly reviewed in [58]).

Firstly, *α*-defensins can induce migration of adaptive immune cells. HNP1 chemoattracts T cells [59–61] and immature DC, although data is conflicting on this point, with one report showing an increase in migration of naïve CD4^+^ T cells only [61] and another showing memory cell migration was also increased [60]. Stimulation of lung epithelial cells with HNP leads to increased adhesion of CD4^+^ T cells [62] and production of IFN-*γ*, IL-2, and IL-8. Adhesion is promoted by increased expression of CD28 and LFA1 on the T cells enabling increased binding to epithelial CD80, CD86, and ICAM1.

Once they are in close proximity, HNP1 can induce pDC to produce IFN-*α* [63] via degradation of I-*κ*B*α* and nuclear translocation of IRF1. Similar signalling is seen in CD4^+^ T cells; HNP induces translocation of NF-*κ*B p50 to the nucleus and induced degradation of I-kB [62]. HNP1 also promotes an increase in the costimulatory molecules CD80, CD86, and CD40 on human monocyte-derived DC [64] as well as maturation markers. In a mixed lymphocyte reaction, DC treated with HNP1 increased T cell proliferation.

Interestingly, there are concentration-dependent outcomes following the exposure of human monocyte-derived DC to *α*-defensins [65]. Low-medium doses of HNP1-3 (up to 1 *μ*g/ml) led to a slight increase in CD86 and HLA-DR expression, while higher doses inhibited the expression of these molecules. Likewise, low dose increased DC production of IL-1*β*, TNF, IL-12p40, and IL-10, while higher doses inhibited this strongly. Consequently, low dose of HNP1-3 increased the DC stimulation of T cell proliferation, and high dose reduced this.

#### 4.1.3. Neutrophil Elastase

Neutrophil elastase (ELANE, NE) is a potent serine protease with broad specificity stored in the primary azurophil granules alongside MPO and *α*-defensins. However, unlike the others, elastase shows mostly anti-inflammatory and regulatory roles (aside from its bacterial-killing capabilities).

Neutrophils transmigrating to sites of inflammation secrete elastase [66] which suppresses later SDF-1—induced T cell transmigration (but not subsequent neutrophil migration). This suppression was found to be elastase dependent.

In macrophages, elastase cleaves TLR2, TLR4, and MD-2 [67], suppressing proinflammatory cytokine production and impairing host defence in a pneumonia model. We do not yet know whether a similar phenomenon occurs with DC, but elastase does induce human immature DC—but not fully mature cells—to shut off IL-6 production and increase TGF-*β* [68, 69]. In a mixed lymphocyte reaction, DC treated with elastase showed a twofold increase in regulatory T cell generation compared to control-treated cells [69] and an overall suppression of lymphocyte proliferation [68]. What is interesting in this study was that DCs treated with primed neutrophil supernatant—that collected following IL-8 priming and albumin-induced degranulation—also induced an increase in Treg in a TGF-*β*-dependent fashion. Likewise, elastase—and, more generally, supernatant from sputum isolated from patients with chronic obstructive pulmonary disease and Cystic Fibrosis—suppressed the DC expression of costimulatory markers, the latter in an elastase-dependent manner [70]. It remains to be explained how elastase can suppress DC responses and induce regulatory T cells, when in these contexts—sputum, or whole neutrophil supernatant—it is present alongside so many proinflammatory mediators.

However, two pieces of work do suggest a proinflammatory role for elastase. IFN-*γ* producing T cells specific for neutrophil elastase have been identified [71]. These T cells were shown to be present in 40-60% of healthy individuals without any history of autoimmune disease. In addition, Souwer and colleagues [72] show that elastase is required for the generation of Th17 responses, owing to its cleavage of DC-produced CXCL8 into a more potent form. How this paper fits into the literature on elastase is complicated; it is in agreement with the proinflammatory nature of NETs, which contain elastase (see Section 4.2 'NETosis') but not with the previous research using elastase in isolation. This incongruence may be a result of the populations of T cells being used in this paper being highly pure, something that is often not the case with older studies.

#### 4.1.4. Cathelicidin LL-37

The cathelicidin LL-37 is a 4 kDa cationic amphipathic peptide stored in secondary granules as the inactive precursor hCAP-18 before being cleaved by proteinase 3 during degranulation. Although it has long been known as a powerful antimicrobial, antifungal, and antiviral agent, now we also recognise its profound immunomodulatory roles.

Cathelicidin is one of the granule peptides most investigated for its role in adaptive immunity, with many influences on DC and T cells described. Interestingly, and reflecting data seen with MPO and elastase, results are split between pro- and anti-inflammatory outcomes (see Table 1), suggesting either concentration-dependent effects or that it acts in concert with other local mediators to produce dichotomous results. During inflammatory disease, LL-37-specific T cells exist; up to 75% of patients with severe psoriasis have T cells recognising LL-37, and these T cells are pathogenic and produce proinflammatory cytokines [73]. T cells from patients with psoriasis proliferate and produce IFN-*γ* in response to ex vivo LL-37 exposure, while T cells from healthy control donors do not. Interestingly, LL-37-specific T cells were seen more frequently in patients with severe disease than with mild clinical scores. A different study [74] showed increased proliferation in healthy control CD4^+^ T cells with LL-37 exposure—this occurred in the presence and absence of DCs. Similarly, delivery of murine cathelicidin (mCRAMP) to *ApoE^−/−^* mice induced activated degranulating CD8^+^ T cells with increased proliferation and IFN-*γ* production [75]. CD4^+^ T cells migrate towards LL-37 [76, 77] and whole peripheral blood leukocytes towards murine cathelin-related antimicrobial peptide (CRAMP) [78] in a dose-dependent fashion, but interestingly CD8^+^ T cells do not [77].

LL-37 can bind DCs directly (a review of the peptide's receptors is included in [79]), inducing maturation and CD86 expression on DCs [80]; this subsequently primes Th1 responses and induces the production of IFN-*γ*. This occurs via binding to the G protein-coupled receptor FPR2 [81].

A prominent role of LL-37 is to form complexes with self-DNA and allow its take up by plasmacytoid DC (pDC). LL-37-DNA complexes move into the endocytic pathway of the DC and therefore promote a strong IFN-*α* response [82]; this pDC type 1 interferon production then matures cDC and leads to pathogenic proliferation and activation of Th1 cells [83]. The same phenomenon—LL-37-facilitated priming of pDC—is seen with bacterial DNA and CpG, allowing rapid responses to microbes [84]. Coexposure to LL-37 and CpG DNA induced the expression of costimulatory molecules CD86 and CD40 and production of IL-6 within five-minute exposure; this was specific, as T cells did not respond to CpG-LL-37 complexes.

*In vivo*, injection of CRAMP alongside OVA led to an enhancement of immune responses—proliferation of splenocytes, IL-4, IL-6, IL-10, and IFN-*γ* production—without skewing of responses [78]. Finally, the proinflammatory role of LL-37 has been targeted in cancer therapy. In a model of mice challenged with SP2/0-CSFRj6-1 tumour cells, LL-37 enhanced vaccination with a DNA vaccine against M-CSFRj6-1. Addition of LL-37 to the vaccine improved the cytotoxic T cell response, IFN-*γ*, and IL-4 production and resulted in prolonged survival [85]. In summary, therefore, there are a number of profoundly proinflammatory outcomes following LL-37's interaction with DC and T cells.

One intriguing study shows that LL-37 is not uniformly proinflammatory but is more discriminating in its immunomodulation. Nijnik et al. [86] showed that LL-37 inhibited IFN-*γ*-induced priming of DC, monocytes, and macrophages, abolishing TNF production following IFN-*γ* exposure. In combination with IFN-*γ*, LL-37 suppressed TNF production following LTA stimulation but not that resulting from Pam3Csk4, flagellin, or NOD2.

Similarly, an increasing body of literature shows LL-37 can also be selectively anti-inflammatory. It specifically inhibits TLR4 ligand signalling in DCs, with no effect on TLR2 signalling [87]. This, in the context of a murine model of allergic contact dermatitis, means that LL-37 has an anti-inflammatory role. In this paper, LL-37 prevented the DC production of IL-6, IL-8, IL-10, and TNF following LPS treatment, but not IL-1*β* or GMCSF; this was via prevention of aggregation of the membrane receptor complex required for signal transduction.

Human DC react similarly to mouse; LL-37 inhibits monocyte-derived DC priming by LPS and subsequent stimulation of T cell proliferation [88]. IL-6 and TNF were totally inhibited by a physiologically relevant dose of LL-37. However, in this study, the signalling by TLR2 and TLR5 ligands (LTA and flagellin) was also inhibited by LL-37.

Direct effects on murine T cells show that it may induce apoptosis of certain cells [89, 90], in particular T regulatory cells and cytotoxic CD8^+^ T cells, which are significantly more sensitive to LL-37 than CD4^+^ cells. However, doses of LL-37 used in these studies were high (up to 100 *μ*g/ml). Murine cathelicidin (mCRAMP) also prevents the development of Th2 responses *in vitro*; isolated T cells from mice lacking cathelicidin overexpress Th2 *in vitro* while Th1 responses are normal [91].

#### 4.1.5. Lactoferrin

Lactoferrin is an 80 kDa protein from the transferrin family present alongside LL-37 in the secondary granules of neutrophils; it is abundant in milk and has strong antimicrobial activity.

When immature human DCs are incubated with lactoferrin, it induces an increase in maturation (CD83), activation (CD80, CD86), proinflammatory cytokine production (with discrepancies between reports), and CCR7 expression (resulting in migration towards CCL21) [92–94]. Consequently, T regulatory cell development is decreased and Th1 cells enhanced. These effects on DC required TLR2 and TLR4 signalling [93].

Used as an adjuvant during sheep red blood cell induced delayed type hypersensitivity, lactoferrin enhances inflammation and increases TNF, IL-10, and MIP1*α* production by total peritoneal cells [95], with subsequent increased lymphocyte proliferation. This is however using a very high dose (>50 *μ*g/ml).

Interest in using bovine lactoferrin as an adjuvant led to its use alongside *Mycobacterium bovis* Calmette-Guerin (BCG). BCG-infected DCs which had been exposed to lactoferrin showed an increase in activation and costimulation markers and increased stimulation of Th1 cells [96]. Use in this system led to a substantial increase in CD4^+^ splenic T cells producing IFN-*γ* [97] compared to BCG alone, ultimately leading to a reduction in *Mycobacterium tuberculosis-*induced pathology [98].

Coculture of neutrophils and CD4^+^ T cells results in the transfer of lactoferrin to the T cell surface [99]. Using the Jurkat cell line, lactoferrin was shown to induce tyrosine phosphorylation in the T cells 10 minutes after exposure; this upregulated CD4 expression through MAPK signalling, and potentially therefore CD3 complex signalling capability [100]. In mice expressing functional human lactoferrin, this boost to T cell signalling resulted in increased TNF and IFN-*γ* production and improved bacterial clearance during *Staphylococcus aureus* infection [101]. Lactoferrin also has been shown to have positive impacts on T cell differentiation and maturation; it promotes CD4 expression and maturation of CD4^+^ cells from precursors and enhances proliferation [102, 103]. Oral administration of bovine lactoferrin boosts CD4^+^ and CD8^+^ T cell numbers in the intestine [104]. Administration of lactoferrin following cyclophosphamide-mediated depletion restores T cell populations in mice [105]. Peripheral blood and splenic lymphocyte populations were significantly restored, particularly CD4^+^ T cells [105]. These interesting results, *in toto*, suggest that lactoferrin could be used to boost CD4^+^ T cells in immunosuppressed individuals.

A truncated lactoferrin peptide comprising the first 11 residues from the N-terminus, hLF1-11, is of interest as a novel antimicrobial. Exposure of differentiating DC to hLF1-11 increases their maturation and their ability to phagocytose *Candida albicans* and subsequently produce more IL-6 and less IL-12p40 [106]. This led to increased generation of Th17 cells.

Lactoferrin applied to pig small intestine mucosal explants [107] is rapidly taken up by lamina propria T cells, and administration of oral recombinant human lactoferrin results in the activation and proliferation of intestinal T cells [108], particularly CD8^+^ T cells which showed enhanced IFN-*γ* production and improved responses to implanted tumours. However, lactoferrin has well-known anti-inflammatory role in mouse and rat models of colitis [109, 110], and this appears to be a result of it decreasing Th1 and Th17 cells in the mesenteric lymph nodes and intestinal lamina propria and skewing towards T regulatory cells instead [111]. This may be a consequence of lactoferrin binding pathogen-associated molecular patterns (PAMPs) such as LPS and therefore dampening down immunity in the gut rather than directly acting on the T cells. However, the possibility remains that in the generally immunosuppressive environment of the intestinal mucosa, the signals generated in T cells by lactoferrin result in T regulatory cell development and suppression of immunopathology. The route of supply of lactoferrin also may have an impact, with single buccal dose and continual diet dosing increasing Th2 responses in the gut, while gastric intubation for delivery increased Th1 cytokine production [112].

Some other work has shown an inhibitory role of lactoferrin on T cell responses. It inhibits proliferation of T cells in an MLR [113] and inhibited proliferation and cytokine production of a Th1 cell line (but not Th2) [114]. Likewise, the transfer of lactoferrin to CD4^+^ T cells [99] induces suppression of IFN-*γ* and enhancement of IL-10 in the activated T cells, suggesting this is another possible route of neutrophil-induced T cell regulation.

#### 4.1.6. Arginase-1

Arginase-1 is present in the tertiary (gelatinase) granules; however, it is inactive at physiological pH unless activated by factors released by primary granule release [115]. Once released, it converts arginine to L-ornithine; as arginine is required for the expression of the T cell receptor zeta chain and subsequent proliferation of T cells [116, 117], the depletion of arginine results in cell cycle arrest and suppression of T cell responses; this is also mediated via an inability to upregulate cyclin D3 [118]. In addition, arginine is necessary for the dephosphorylation of cofilin, which stabilises the immunological synapse which forms between T cells and DC; arginase therefore destabilises the synapse and prevents adequate T cell activation [119, 120].

Arginase release from neutrophils is uniformly suppressive to T cell responses, unlike the more nuanced results for other granule mediators.

In addition to infection and sites of purulent inflammation [117], this suppression of T cell responses via arginase release occurs during pregnancy; neonatal neutrophils have higher arginase content and are more suppressive than adult neutrophils [121]. This is an intriguing explanation proposed for why newborns are susceptible to infection. Activated neutrophils are also present in the spleen of poststroke mice [122], and these produce arginase, resulting in suppressed T cells as a consequence of decreased CD3 zeta; similarly, neutrophils from airway neutrophils isolated from patients with Cystic Fibrosis suppress T cell activation via arginase [123].

Myeloid-derived suppressor cells produce arginase, which is one of the most prominent explanations for their suppressive activity; however, much of the research described above did not unpick relative contributions of MDSC and suppressive neutrophil activities or which subsets of neutrophils were involved (see Section 5 - a note on immature and low-density neutrophils).

#### 4.1.7. Gelatinase

Gelatinase, a 94 kDa type IV collagenase [124], is present in the tertiary granules of neutrophils. Research into the effects of gelatinase on T cell responses has not been examined in detail. However, recently, it has been shown that gelatinase released from neutrophils is indispensable for the generation of contact hypersensitivity responses [125]. In this interesting paper, neutrophil depletion reduced DC migration into the site of sensitization (the ear) and subsequent allergen-specific T cell priming and Th1 development. This is intriguing as gelatinase and arginase are released from the same granules but appear to have opposite impacts on adaptive immunity.

#### 4.1.8. Cathepsin G

Cathepsin G is a member of the serine protease family and is predominantly found in primary neutrophil granules, although it has also been detected in various myeloid cells and APCs [126, 127].

Cathepsin G can serve as a signal that amplifies the inflammatory response. For example, it can act as a chemoattractant for neutrophils and monocytes [128]. Wittamer et al. revealed a role for cathepsin G in the processing and maturation of chemerin, a chemoattractant that specifically attracts APCs [129]. In addition, it is responsible for the quantum proteolytic processing of CXCL5 and CCL15 in to more potent chemotactic factors [130].

Cathepsin G is capable of binding to human lymphocytes and acting as a chemokinetic stimulant of T cells [128, 131]. The administration of cathepsin G in mice, together with antigen, enhances the antibody response and increases IFN*γ* and IL-4 production [132]. Furthermore, cathepsin G plays a critical role in proinsulin processing and the activation of diabetogenic T cells [133]. The downregulation of cathepsin G by specific inhibitors or siRNA was shown to mitigate the activation of CD4^+^ T cells in nonobese diabetic (NOD) mice, leading to reduced blood glucose and improved function of islet *β* cells [134].

However, cathepsin G has also been implicated in the regulation of excessive inflammation. For instance, it can cleave the IL-1-related alarmins IL-18 and IL-33, as well as IL-15, which is important for both T cell and NK cell homeostasis [135]. In addition, cathepsin G has been shown to disrupt CCL5 and CCL3 chemokine gradients, both of which recruit T lymphocytes [136, 137]. It also reduces the bioactivity of the T cell-stimulating cytokines IL-2 and IL-6 at sites of inflammation and catalyzes the shedding of their receptors, thereby enhancing its suppressive effects [138].

In Cystic Fibrosis patients, cathepsin G can cleave the surface antigens CD2, CD4, and CD8, resulting in a temporary functional impairment of T lymphocytes and a state of immunological unresponsiveness in inflammatory foci [139]. Kish et al. also recently showed that during hapten skin sensitization, neutrophil cathepsin G inhibits the production of IL-12 from hapten-presenting DCs. This in turn suppresses the development of hapten-reactive CD4^+^ T cells and their differentiation in to IFN*γ*-producing effector cells [140].

#### 4.1.9. Cathepsin D

Cathepsin D is an aspartyl protease, ubiquitously distributed in lysosomes. It has been implicated in a wide variety of biological processes, including proteolytic degradation and apoptosis, as well as various inflammatory disorders.

Cathepsin D has been suggested to play an essential role in tissue homeostasis. For example, it is required for the survival of mice beyond 4 weeks [141]. Mice deficient for this enzyme exhibited progressive atrophy of the intestinal mucosa and profound destruction of the lymphoid organs and cells [141]. Moreover, ceramide activates lysosomal cathepsin D (and B) to attenuate autophagy and induce ER stress to suppress myeloid-derived suppressor cells [142].

Cathepsin D is also involved in class II MHC-restricted antigen processing and the generation of T cell epitopes. This was first shown in 1994 when M. van Noort demonstrated that cathepsin D could induce the release of T cell stimulatory fragments from hen egg white lysozyme (HEL) *in vitro*. Cathepsin D has since been implicated in the proteolytic processing of thyroglobulin, myoglobulin, and tetanus toxin [143–145]. While not in neutrophils, DC cathepsin D is required for lipid antigen presentation by DCs: ligand activation of PPARg (Peroxisome Proliferator-Activated Receptor g) upregulates cathepsin D, which in turn generates the mature form of saposins, lipid transfer molecules that facilitate lipid loading to CD1d molecules [146].

On the other hand, cathepsin D has also been implicated in apoptosis and the resolution of innate immune responses. Caspase 8 is directly activated by cathepsin D, which in turn triggers a proapoptotic pathway in neutrophils. Inactivation of cathepsin D by both genetic and pharmacological means delayed neutrophil apoptosis and prolonged the inflammatory response to LPS *in vivo* [147]. Bidere et al. also demonstrated that cathepsin D triggers a rapid conformational change in Bax upon apoptotic signalling. This induces the release of mitochondrial apoptosis-inducing factor (AIF), which controls the early apoptotic phenotype in activated T lymphocytes [148].

### 4.2. NETosis

Neutrophils release extracellular fibres known as neutrophil extracellular traps (NETs, [7]). NETs comprise cytoplasmic and granule proteins bound to a web of chromatin, and their expulsion encapsulates and kills microbes. Notably, murine neutrophils produce NETs more slowly and less efficiently than human neutrophils; however, these differences could be attributed to murine neutrophils being isolated from the bone marrow, while human neutrophils are peripherally derived [149].

NETosis can be triggered by a wide range of stimuli including, *in vitro*, biochemical agents, pathogens (for example, *Staphylococcus aureus)*, and their products (for example, LPS), although PMA (phorbol 12-myristate 13-acetate) is used most often as it potently induces NETting in all studies [150–152].

Neutrophil products, including granule contents, embedded within NETs can cause host tissue damage and disease [153]. The first study to describe NETs analysed their composition using immunofluorescence and noted DNA, histones, neutrophil elastase (NE), cathepsin G, myeloperoxidase (MPO), lactoferrin, and gelatinase [7]. This suggested a network of DNA decorated with many granule proteins. A more recent proteomic approach analysing PMA-induced NETs identified 24 NET-associated proteins, including granule proteins in addition to cytoplasmic proteins such as calprotectin, and cytoskeletal proteins actin *β*/*γ* [154]; the most abundant nonhistone protein was NE. Of importance for interpretation, the proteomic analysis did not find previously described NET-associated proteins H1, BPI, cathelicidin (hCAP-18), and pentraxin 3 (PTX-3). Subsequent immunoblot analysis established the presence of BPI, but not of PTX-3 or hCAP-18. They propose that these proteins perhaps are loosely attached to NETs and therefore were lost during the isolation procedure [154]. It has also been suggested that different stimulants can induce varying NET composition.

Many NET experiments are performed following induction of NETosis with PMA, which also profoundly activates T cells. Although attempts are made to wash NETs and control for this, it must be borne in mind when interpreting the impact of NETs on adaptive immunity. New methods of inducing NETs without PMA [151] are now defined, and use of these will allow confirmation of current results.

The presence of antimicrobial peptides in the NET structure licences the DNA to become immunomodulatory and to activate DCs. NETs stably interact with DCs, unlike apoptotic or live neutrophils [155], and the DCs take up NET components, notably proteinase 3 and myeloperoxidase. NETs induce type one interferon production by pDCs, a first step to autoimmunity [156], and promote Th1 responses in the mouse model collagen-induced arthritis [157] through enhancing DC maturation and costimulation. In the rheumatoid joint, NETs containing citrullinated peptides are taken up by fibroblasts, which then present NET peptides to local T cells [158]; this leads to damage of local cartilage and development of autoimmune populations of T cells. NET fragments (following digestion with DNAse) induce CD40, CD80, CD86, and MHC II upregulation on mouse bone marrow-derived DCs via TLR-MyD88 signalling [159]; this led to proliferation of allogeneic CD4^+^ T cells. This is important as DNAse is suggested as a therapy for many diseases and is approved for use in Cystic Fibrosis. NETs are triggered by cigarette smoke, and these drive pDC maturation and activation [160]. NETs and cigarette smoke extract together induced pDC to upregulate CD40, CD86, and MHC II and to produce IFN-*α*, IL-6, and IL-12p70. As a result, these pDCs produced more inflammatory cytokines (IFN-*γ* and IL-17), consequent to increased Tbet and ROR*γ*t, than pDC exposed to air control. This is proposed to be one way in which inflammatory responses are generated and maintained in COPD.

NETs are released by patients with SLE complex self DNA and the granule peptides LL-37 and HNPs; these NET-DNA complexes triggered TLR9 and promoted pathogenic type 1 IFN release [161].

In addition to priming DCs, NETs can act directly on CD4^+^ T cells. Human T cells reacted to NETs by increased phosphorylation of the tyrosine kinase ZAP70, which is critical for downstream T cell activation [162]. This led to increased expression of the activation markers CD25 and CD69. Mechanistically, it appears that the presence of NETs primes CD4^+^ T cells so that they are able to respond to and proliferate following the presentation of low doses of antigen, even in the absence of costimulation. NETs—and neutrophils in general—are most frequently associated with Th17 responses in local T cells, but they can also induce Th2 responses. Recently it has been shown that NETs induced in the lung following rhinovirus infection promote type 2 immunity and exacerbations of asthma [163].

HIV or SIV-infected individuals overproduce NETs. It is suggested that these NETs trap and induce apoptosis in CD4^+^ and CD8^+^ T cells and that this may be one mechanism by which these T cells are lost during infection [164].

In summary, NETs are highly proinflammatory for DC and T cell responses, and the granule peptides coating these NET strands play a large part in this, despite their frequent anti-inflammatory roles when used in isolation. It is possible that increased concentration on the strands licences proinflammatory roles, that the particular combination of proteins acts in concert with unexpected outcomes for T cells, or that the presence of DNA tips the balance from anti- to proinflammatory.

Intriguingly, a regulatory role for NETs is shown in [165]. This paper exposed monocyte-derived DC to human NETs and showed how this downregulated LPS-induced maturation, cytokine production, and costimulatory molecule expression. Unsurprisingly, this had the consequence of reducing subsequent CD4^+^ T cell proliferation [165]. In this paper, NET fragments were induced and isolated with A23187 stimulation then Alu1 restriction enzyme treatment. It is possible that differences in outcome from published NET papers reflect the methods of isolation.

Finally, it is worth noting that not only NETs released by neutrophils impact the adaptive immune response; the cell bodies, cytoplasts, left behind following NETosis are also capable of doing so. In asthma, it has been shown that these cytoplasts were present in draining lymph nodes and induced DC to promote Th17 generation [166]. This exciting paper opens up a whole new area of research as we consider how neutrophils can affect T cells in lymph nodes.

## 5. A Note on Immature and Low-Density Neutrophils and Myeloid-Derived Suppressor Cells

Isolation of neutrophils using density gradients can allow some neutrophils to come out in the isolated PBMC layer; these low-density neutrophils impair T cell proliferation *in vitro* and may represent granulocytic myeloid-derived suppressor cells (MDSC), although the low-density layer may also include more than one population [167, 168]. It is not currently possible to fully distinguish myeloid-derived suppressor cells from mature neutrophils [32], both being CD14- CD15+ CD11b+ CD66b+ HLADR- [32]. Furthermore, many of the studies in this review were performed before the era of MDSC, and as such it is not possible to unpick relative impacts of MDSC vs. activatory neutrophils in each case.

Recently, CD10 has been identified as a marker which discriminates between mature (CD10+) and immature (CD10-) neutrophils, with the former inhibiting T cells via arginase-1 and the latter promoting T cell proliferation and IFN-*γ* production [168]. Increasing the use of this marker in flow cytometry panels may enable discrimination of these subsets and examination of their impact during inflammatory disease. It remains an intriguing possibility that the dichotomous results presented at the beginning of this article, from *in vitro* and *in vivo* experiments over many decades, may include results from soon-to-be clearly delineated subsets of cells—and that we may soon be able to examine them with fresh eyes.

Overall, what this means is that it is essential for researchers to examine and present neutrophil maturation status and gross phenotype clearly, as well as clearly describing isolation methods used, so that readers are in full knowledge of the facts.

Finally, neutrophils can interact with Dynabead-style activation beads which are commonly used to activate T cells [167], leading to suppression of T cell proliferation *in vitro* which does not reflect *in vivo* situations. This must be considered when reflecting on published data.

## Figures and Tables

**Table 1:** Abundant contents of neutrophil granules and their impact on T cell immunity.

Mediator	Granule	Effect on DC/T cells	Outcome	Species	Reference
MPO	**Primary**	Is a T cell antigen	**Activatory**	Human, mouse	[47, 48]
		Induces IL-17A production, due to being an autoantigen	**Activatory**	Mouse	[51]
		Suppresses lymphocyte proliferation	**Suppressive**	Human	[54]
		Suppresses CD4^+^ T cell responses	**Suppressive**	Mouse	[55]
		Suppresses DC activation and cytokine production	**Suppressive**	Mouse, human	[53, 55]
		MPO^−/−^ mice have enhanced T cell responses in arthritis	**Suppressive**	Mouse	[55]
		MPO^−/−^ mice have increased T cell responses in nephritis	**Suppressive**	Mouse	[52, 53]

HNPs	**Primary (only human)**	Low dose: matures, activates, and induces cytokine secretion by DC	**Activatory**	Human	[65]
		Chemoattracts naïve but not memory T cells	**Activatory**	Human	[61]
		Chemoattracts naïve and memory T cells	**Activatory**	Human	[60]
		Are produced by monocyte-derived DC	**Activatory**	Human	[118]
		HNP1 induces pDC to produce IFN*α*	**Activatory**	Human	[63]
		Enhances T cell adhesion to lung epithelium	**Activatory**	Human	[62]
		Induces NF-*κ*B signalling in CD4^+^ T cells	**Activatory**	Human	[62]
		HNPs are anti-inflammatory to DC	**Suppressive**	Human	[169]
		High dose: inhibits activation, induces IL-8	**Suppressive**	Human	[65]

Elastase	**Primary**	Is a T cell antigen	**Activatory**	Human	[71]
		Strongly promotes Th17 responses via cleavage of DC CXCL8	**Activatory**	Human	[72]
		Induces DC to prime Treg	**Suppressive**	Human	[69]
		Induces DC to produce TGF-*β*	**Suppressive**	Human	[68]
		Suppresses T cell proliferation in an MLR	**Suppressive**	Human	[68]
		Reduces T cell transmigration	**Suppressive**	Human	[66]
		Inhibits DC maturation and suppresses costimulation	**Suppressive**	Human	[70]

LL-37	**Secondary**	Is a T cell antigen	**Activatory**	Human	[73]
		Induces proliferation CD4^+^ T cells (+ PHA)	**Activatory**	Human	[74]
		Is a chemoattractant for CD4^+^ T cells (FPR2 dependent)	**Activatory**	Human, mouse	[61, 77, 78]
		Activates CD8^+^ T cells and induces degranulation	**Activatory**	Mouse	[75]
		Matures DC primes Th1 responses	**Activatory**	Human	[80]
		Complexes DNA and is taken up by pDC	**Activatory**	Human	[82, 84]
		Enhances vaccination against tumour model	**Activatory**	House	[85]
		Induces DC CD86 and migration to CCR7 ligands	**Activatory**	Mouse	[81]
		Boosts T cell proliferation and cytokine production at immunisations	**Activatory**	Mouse	[78]
		Is internalised into cytoplasm and nucleus of DC, inducing CD86	**Activatory**	Human	[170]
		Induces apoptosis Treg	**Activatory**	Mouse	[90]
		Induces apoptosis CD8^+^ T cells	**Suppressive**	Mouse	[89]
		Prevents development Th2 *in vitro*	**Suppressive**	Mouse	[91]
		Suppresses IFN-*γ* activation of DC	**Suppressive**	Human	[86]
		Inhibits DC responses to TLR4 ligands	**Suppressive**	Mouse	[87]
		Inhibits TLR signalling to DC	**Suppressive**	Human	[88]

Lactoferrin	**Secondary**	Is taken up by T cells		Pig	[107]
		Matures and activates DC	**Activatory**	Human	[92, 94, 96]
		Promotes T cell proliferation and Th1 generation	**Activatory**	Human	[92, 94, 96]
		Increases inflammation during DTH response	**Activatory**	Mouse	[95]
		Boosts CD4^+^ IFN-*γ* production in concert with BCG vaccination	**Activatory**	Mouse	[97]
		Upregulates the CD4 molecule	**Activatory**	Human	[100]
		Increases T cell cytokine production during infection	**Activatory**	Mouse	[101]
		Increases intestinal CD8^+^ T cell activation	**Activatory**	Mouse	[108]
		Decreases proinflammatory CD4^+^ cells in intestinal inflammation	**Suppressive**	Mouse	[111]
		Prevents T cell proliferation in an MLR	**Suppressive**	Human	[113]
		Prevents Th1 but not Th2 proliferation	**Suppressive**	Human	[114]

Arginase-1	**Tertiary**	Suppresses T cell proliferation via TCR-zeta	**Suppressive**	Human, mouse	[115, 117]
		Induces cell cycle arrest	**Suppressive**	Human	[118]
		Inhibits development of immunological synapse	**Suppressive**	Human	[120]

Gelatinase	**Tertiary**	Induces DC migration and T cell priming in DTH	**Activatory**	Mouse	[125]

MPO: myeloperoxidase; DC: dendritic cell; HNP: human neutrophil peptide; pDC: plasmacytoid dendritic cell; IFN-*α*: interferon-*α*; NF-*κβ*: nuclear factor-kappa beta; MLR: mixed lymphocyte reaction; PHA: phytohemagglutinin; FPR2: formylpeptide receptor 2; IFN*γ*: interferon-gamma; TLR: toll-like receptor; DTH: delayed-type hypersensitivity.
